# Does Facial Resemblance Enhance Cooperation?

**DOI:** 10.1371/journal.pone.0047809

**Published:** 2012-10-19

**Authors:** Trang Giang, Raoul Bell, Axel Buchner

**Affiliations:** Department of Experimental Psychology, Heinrich Heine University Düsseldorf, Düsseldorf, Germany; CNRS, Université de Bourgogne, France

## Abstract

Facial self-resemblance has been proposed to serve as a kinship cue that facilitates cooperation between kin. In the present study, facial resemblance was manipulated by morphing stimulus faces with the participants' own faces or control faces (resulting in self-resemblant or other-resemblant composite faces). A norming study showed that the perceived degree of kinship was higher for the participants and the self-resemblant composite faces than for actual first-degree relatives. Effects of facial self-resemblance on trust and cooperation were tested in a paradigm that has proven to be sensitive to facial trustworthiness, facial likability, and facial expression. First, participants played a cooperation game in which the composite faces were shown. Then, likability ratings were assessed. In a source memory test, participants were required to identify old and new faces, and were asked to remember whether the faces belonged to cooperators or cheaters in the cooperation game. Old-new recognition was enhanced for self-resemblant faces in comparison to other-resemblant faces. However, facial self-resemblance had no effects on the degree of cooperation in the cooperation game, on the emotional evaluation of the faces as reflected in the likability judgments, and on the expectation that a face belonged to a cooperator rather than to a cheater. Therefore, the present results are clearly inconsistent with the assumption of an evolved kin recognition module built into the human face recognition system.

## Introduction

Theories within evolutionary psychology predict that the human mind comprises mechanisms that serve to facilitate kin-recognition. Distinguishing between close genetic kin and non-kin is essential for cooperation and incest avoidance. Cooperation has long been a puzzle for evolutionary theorists because it often implies accepting costs to help others, which seems inconsistent with Darwin's belief that all organisms strive to increase their own fitness. A major breakthrough in the evolutionary explanation of cooperation came when William Hamilton proposed his theory of inclusive fitness [1], [2]. According to this theory, helping relatives may pay off in evolutionary terms because it implies investing into individuals that share genes with the helper. Therefore, helping behavior that is directed to close kin can support the individual's *inclusive* fitness. Hamilton's law states that helping is profitable when

r>c/b,

where *r* is the degree of kinship between two individuals, *c* is the cost incurred by the helping individual, and *b* is the benefit of the individual who receives help. An implication of this formula is that cooperation increases with the degree of kinship [3]. However, in order to be able to restrict certain forms of cooperation to close kin, it is necessary to discriminate kin from non-kin. “If [an individual] could learn to recognize those of his neighbors who really were close relatives and could devote his beneficial actions to them alone an advantage to inclusive fitness would at once appear. Thus a mutation causing such discriminatory behavior itself benefits inclusive fitness and would be selected” (p. 21f) [4]. Therefore, kin selection theory predicts that people might have evolved kin recognition mechanisms that help them to selectively channel cooperation to close kin.

What psychological mechanism might underlie kin recognition in humans? There is evidence that human kin recognition is based on a number of highly automatic, cue-based kin recognition processes, most of which are also found in other animals [5]. It is well established that proximity during childhood is probably used as a kinship cue in humans. Maternal-perinatal association may increase the effects of this variable [6]. Lieberman and Lobel [7] investigated the effects of living in close proximity during the first years of life in a Kibbutz. Coresidence duration during childhood was found to be correlated with sexual aversion directed towards opposite-sex peers, and it was also positively correlated with the level of altruism directed towards peers, consistent with other studies showing that coresidence duration has positive effects on cooperation [6]. These findings suggest that spatial proximity during childhood may be used as a kinship cue to regulate incest avoidance and kin support. Thus, it is well established that coresidence during childhood and maternal association are associated with incest avoidance and cooperation. Also, humans typically learn explicitly and repeatedly who their relatives are. In the presence of such strong and highly reliable cues to kinship, other cues may be ignored [6].

A second class of kinship recognition mechanisms is based on phenotype matching. There is evidence that both animals and humans use body odor to discriminate between kin and non-kin [8], [9], [10]. In addition to these mechanisms, it has also been proposed that humans use facial self-resemblance as a kinship cue [11], [12], [13]. The closer related two individuals are, the more genes they share, and the more similar they will look. In the present study, we examine the effects of facial self-resemblance on cooperation, liking, and trust. Note that facial resemblance could influence cooperation in two ways. One hypothesis is that we learn the faces of our family members when we grow up. When we encounter similar looking individuals, the emotional reactions towards our family members may affect the emotional evaluation of resembling faces via transference effects [14]. These transference effects may be due to a general tendency to generalize our feelings towards other people and objects to people and objects that resemble them. A second hypothesis is that spotting self-resemblance in a stranger's face might represent a special adaptation that serves to promote kin support. Such an adaptation would only provide an advantage over contextual kinship cues such as verbal communication, co-socialization, childhood coresidence, and maternal-perinatal association when family relationships are unclear (i.e., when paternal uncertainty is high) [15]. Given the comparably low estimates of non-paternity in many human societies [16], [17], it is unclear whether natural selection would favor this mechanism.

Evidence in favor of such a mechanism is mixed. It has been shown that people (especially men) are more willing to help children whose faces resemble their own [18], [19], but the effects are inconsistent [20]. There are also several studies showing that facial self-resemblance facilitates trust and cooperation in social-dilemma games [11], [21]. The most reliable effects of facial resemblance on positive pro-social attributions have been obtained by DeBruine [12], [13], [22], [23] in a paradigm that seems to be deliberately designed to detect even small and subtle effects of facial resemblance on trustworthiness judgments. In most studies, the procedure starts by creating a composite face by morphing the faces of 20 individuals. Presumably, this aspect of the procedure serves to eliminate all distinctive features of the face that might influence pro-social attributions. Then different versions of the same composite face are created–one that is morphed with the participant (the self-resemblant face), and others that are morphed with other participants. The different versions of the face are simultaneously presented, and the procedure forces participants to pick the one that seems most trustworthy. Note that the versions are identical, with the only difference being the self- or-other-resemblance of the faces. Although this paradigm has proven useful for obtaining reliable effects of facial self-resemblance on judgments of trustworthiness [12], [13], [22], [23], it seems like a highly artificial task that is deliberately designed to detect even minimal effects of facial resemblance. One could argue that participants are forced to rely on facial resemblance when making their trustworthiness judgments because all other variables that could have influenced their behavior have been eliminated. Everyday situations are not at all like that. For instance, in most situations there is plenty of information available that could influence the decision of whether to trust somebody or not. If the effects of facial resemblance on cooperation are due to a kin recognition module that has been selected for its beneficial effects on inclusive fitness, then facial resemblance should have pronounced effects on cooperative behavior even when other information is available. Furthermore, often the task is not to pick a cooperation partner from a group of similar individuals, but rather to decide whether to engage in social cooperation with a certain individual or not.

In the present study, our aim was to test whether the effects of facial resemblance generalize to a situation that provides a more realistic assessment of the effects of facial self-resemblance on cooperation. There were several procedural differences compared to the studies of DeBruine described above. First, the faces are presented sequentially. Furthermore, we morphed the participants' faces with real faces instead of facial composites to reduce the artificiality of the stimulus material. There is some evidence that the facial resemblance effects generalize to this paradigm [11], but generally the results are inconsistent. For instance, there are several experiments that examine whether voters show enhanced trust in a politician whose face has been morphed with their own face [24], [25]. Only in one of these experiments an effect of facial self-resemblance on political preference was revealed; in all the other experiments interactions were found which could not be reliably replicated. Although the results were interpreted as evidence for the assumption that voters prefer candidates who resemble them, the findings are highly inconsistent, and a more objective interpretation would be that the hypothesis is only weakly supported.

To examine the effects of facial resemblance on cooperation and trust, we applied a paradigm that has been shown to be very sensitive to cues of facial trustworthiness in a previous study [26]. Participants played a cooperation game in which they could invest money into a joint business venture with virtual interactants who either cooperated or cheated. In a surprise test phase, participants saw the faces together with new faces, and were asked to rate the likability of the faces. Next, they classified the faces as old or new. When a face was classified as old, they were asked to indicate whether the face belonged to a cheating or cooperative interactant. This paradigm has proven to be a useful tool in the study of cooperation [26], [27]. Participants' behavior in this paradigm is strongly affected by properties of the stimulus faces that are known to affect trust and cooperation. For instance, it is known that facial trustworthiness is strongly associated with the overall positive or negative evaluation of a face [28]. Consistent with this finding, the a-priori likableness of the faces (that had been assessed in a norming study) was positively associated with (a) the willingness to invest into the cooperation game, (b) the emotional evaluation of the face as expressed in the likability ratings, and (c) the tendency to guess that a face belonged to a cooperator in the source memory test. All of these dependent variables were also affected by the facial expression of the faces. Smiling, relative to an angry facial expression, was associated with enhanced cooperation and likableness of the faces, and was also associated with benevolent guessing in the source memory test [26]. What is more, likability ratings and guessing biases were also affected by the pre-normed trustworthiness of the faces. These findings prove that the paradigm applied in the present study is highly sensitive to manipulations of facial trustworthiness.

Based on the assumption that facial self-resemblance enhances trust and cooperation, we predicted that these findings should be replicated using facial self-resemblance–manipulated by morphing the participants' own faces with other faces–as the independent variable. Specifically, we predicted that facial self-resemblance would be positively associated with (a) the willingness to invest into the cooperation game, (b) the emotional evaluation of the face as expressed in the likability ratings, and (c) with benevolent guessing in the source memory test. In addition to its effects on a potential kin recognition mechanism, facial self-resemblance may also have beneficial effects on memory simply because it is an extremely well known stimulus feature to which people have been exposed very often during their lifetime. For highly overlearned faces such as one's own face, people may have developed rich representations [29] that may facilitate face identification, face processing, and face recognition [29], [30]. Enhanced memory for familiar faces has also been demonstrated for other super-familiar faces of family members, friends, and celebrities [31], [32]. Expertise may also explain why people are better at recognizing faces of their own race, sex, and age [33], [34], [35], [36]. Based on the assumption that super-familiarity facilitates face recognition, superior old-new recognition of self-resembling in comparison to other-resembling faces can be expected.

## Methods

### Ethics statement

The study was approved by the ethics committee of the medical faculty of Heinrich Heine University Düsseldorf. Participants signed an informed consent before participating in the experiment.

### Participants

The original sample consisted of 66 students at Heinrich Heine University Düsseldorf aged 18 to 30 years. Only people without facial hair were allowed to participate in the study. Participants who did not match the inclusion criteria were offered to participate in another study. Participants were matched with yoked partners of the same sex who participated in the experiment on another day. Four women and their yoked partners had to be excluded from the data analysis because two women recognized their own face, one was interrupted by a fire alarm, and one failed to attend the second session. The remaining sample consisted of 58 white adults (40 female, 18 male; mean age  = 21.36, *SD* of age  = 2.96). 82.76% of the participants had at least one sibling.

### Materials

A total of 80 male and 80 female white stimulus faces with neutral expressions were taken from face databases [37], [38], [39]. All photographs had the same size (400×499 pixel grayscales). Each photograph showed a face on a white background. To avoid morphing artifacts, only faces without facial hair were selected. Half of the male and female faces were assigned to two sets of 40 male and 40 female photographs each (henceforth Set A and B). All faces were edited to have the same brightness.

The photographs of the participants' faces were taken approximately 1 week before the proper experiment. Light conditions and the size of the faces were held constant. All participants had a neutral facial expression when photographed. Participants were asked to remove their glasses, piercings, and makeup, which could have interfered with the morphing process. Scars and birth marks were removed digitally (using Photoshop CS5). Participants were told that the photograph would be used to show it to other participants in the cooperation game.

**Figure 1:**
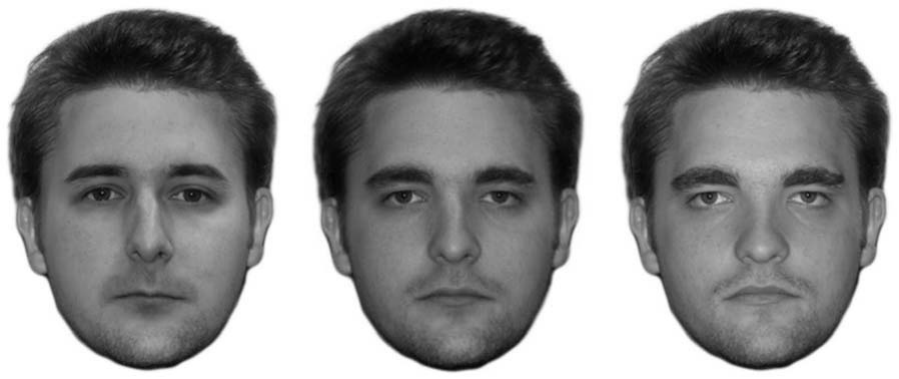
Example morph. On the left side, the participant's face (eye brows, eyes, nose mouth) is shown that has been pasted into the shape of the stimulus person's face. On the right side, an example for a stimulus face is shown. In the center, the face morph is shown (morph ratio = 40:60). Both faces are used for illustration purposes only (i.e., because of copyright restrictions, the faces do not correspond to faces actually used in the present study). Written consent (as outlined in the PLoS consent form) was obtained from both individuals before publishing these photos.

Each participant's face was morphed with all persons of the same sex in either Set A or B. One participant and his or her yoked partner formed a pair. One member of each pair was randomly assigned to either Set A or B; the other member was assigned to the remaining set. Each participant's face (i.e., eye brows, eyes, nose, and mouth) was cut out, edited to have the same brightness as the stimulus face, and pasted into the shape of the stimulus face. Then the participant's face was morphed with the stimulus face using Abrosoft FantaMorph Deluxe 5.2.5 (see Figure 1), which had been used in a previous study [40]. Similar to previous studies [11], [21], [24], [25], the participants' faces and the stimulus faces were blended in a 40∶60 ratio to create the self-resembling face (i.e., a morph consisted of 40% of the participant's face, and 60% of the stimulus face). We morphed only the inner face of the participants to ensure that superficial similarities (e.g., in the participants' hairstyle or clothes) between the stimulus faces and the participants could not influence the results, and to avoid artifacts of the morphing procedure.

### Norming study

#### Method

To ensure that we successfully manipulated self-resemblance, we conducted a norming study in which 25 students who did not participate in the proper experiment rated the similarity and the perceived degree of kinship between the participants' faces and the morphed stimulus faces. For comparison, we selected photographs of first degree relatives from the KinFace database [41], [42], which provides 200 photographs of celebrities in young adult age and the same number of facial photographs of first-degree relatives (parent or child) photographed at approximately the same age. To rule out that knowledge about the celebrities influenced the results, we selected only faces of white parent-child pairs that were not recognized by four independent raters (half of which were female). Sixteen father-son pairs, and four mother-daughter pairs were selected based on these criteria. These twenty parent-child face pairs were compared with sixteen male and four female participant-morph face pairs that were randomly selected from the whole pool of available participant-morph face pairs by a computer program.

All faces had the same size (228×265 pixel grayscales). The faces were edited so that only the inner face (eye brows, eyes, nose, and mouth) was shown on a white background. For each participant in the norming study, half of the twenty faces of the KinFace database (8 male and 2 female faces), and half of the twenty faces of the participants (8 male and 2 female faces) were presented together with matching faces (i.e., with first-degree relatives from the KinFace database, or with self-resemblant faces). The other half of the faces were presented with non-matching faces from the same sources.

First, participants were asked to indicate whether they knew one of the faces. Then, participants were asked to judge the resemblance of the faces on a scale ranging from 1 (“very dissimilar”) to 6 (“very similar”). Finally, participants were asked to rate the perceived degree of kinship of the two faces on a scale ranging from 1 (“not related”) to 6 (“closely related”). Nine participants indicated to know at least one of the shown faces. To rule out that knowledge about the faces influenced the results, these nine participants were excluded from further data analysis.

#### Results

**Figure 2:**
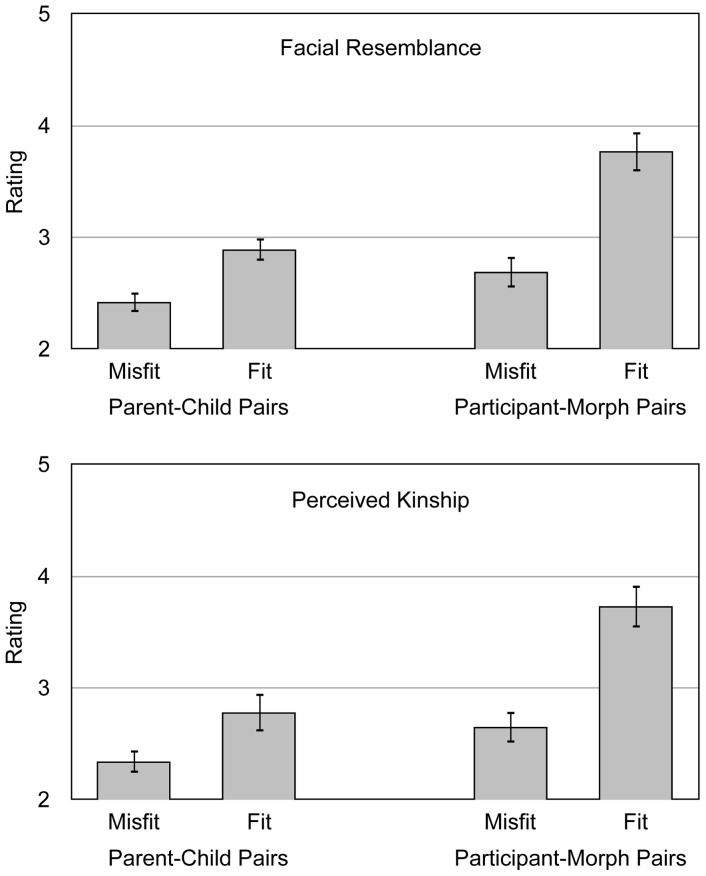
Results of the norming study. Mean ratings for mismatching and matching parent-child face pairs (taken from the KinFace database) and participant-morph face pairs. Upper panel: Mean facial resemblance ratings on a scale ranging from 1 (“very dissimilar”) to 6 (“very similar”). Lower panel: Mean ratings of the perceived degree of kinship on a scale ranging from 1 (“not related”) to 6 (“closely related”). The error bars represent the standard errors of the means.

The results are shown in Figure 2. A 2×2 MANOVA showed that the facial resemblance ratings differed as a function of match, *F*(1,15) = 53.72, *p*<.01, *η* ^2^ = .78, and database, *F*(1,15) = 30.54, *p*<.01, *η* ^2^ = .67. Most importantly, there was also a significant interaction between the two variables, *F*(1,15) = 14.78, *p*<.01, *η* ^2^ = .50. Matching parent-child pairs were perceived as being more similar than non-matching face pairs taken from the KinFace database, *t*(15) = 4.26, *p*<.01, *η* ^2^ = .55. However, the difference in facial resemblance between matching and mismatching face pairs was even more pronounced for participant-morph pairs used in the present study, *t*(15) = 7.21, *p*<.01, *η* ^2^ = .78.

It seems especially interesting whether people used facial resemblance to estimate the degree of kinship between the individuals depicted. The kinship ratings were significantly affected by match, *F*(1,15) = 50.31, *p*<.01, *η* ^2^ = .77, and database, *F*(1,15) = 41.99, *p*<.01, *η* ^2^ = .74. These main effects were qualified by a significant interaction between match and database, *F*(1,15) = 11.21, *p*<.01, *η* ^2^ = .43. Consistent with previous results showing that people can detect kinship among others based on facial resemblance [15], [43], [44], first-degree relatives of the KinFace database were correctly judged to be more closely related than mismatching pairs of faces from the same source, *t*(15) = 3.43, *p*<.01, *η* ^2^ = .44. However, the difference between matching and mismatching face pairs was even more pronounced for participant-morph face pairs, *t*(15) = 6.95, *p*<.01, *η* ^2^ = .76. Matching participant-morph face pairs were judged to be more closely related than parent-child pairs from the KinFace database that were actually closely related, *t*(15) = 6.08, *p*<.01, *η* ^2^ = .71.

The norming study clearly shows that facial similarity was successfully manipulated. Even more importantly, facial resemblance had pronounced effects on the perceived degree of kinship between the participants of the proper experiment and the morphs. Participants and morphs were perceived to be even more closely related than actual first-degree relatives. One might criticize that the estimation of the resemblance and perceived kinship of actual first-degree relatives might be underestimated due to paternal uncertainty. However, recent studies show that the prevalence of non-paternity in Western societies is so low (about 1%) that it can be considered irrelevant for the comparison reported above [16]. Thus, if a kinship recognition mechanism exists that is sensitive to facial resemblance, one would predict that it should be activated by the manipulation of facial resemblance used in the present study.

### Procedure

Participants attended two sessions. In the first session, which took about ten minutes, the participants were informed that their photograph would be shown to other participants in a social-cooperation game in which they would also take part in the second session. The second session was scheduled to occur about one week later and lasted about 45 minutes. The participants played the social-cooperation game and took part in a surprise source monitoring test.

#### Social-cooperation game

In the first part of the experiment, participants played the social-cooperation game successfully used by Bell et al. [26] to examine the effects of facial likability and facial expression on trust and cooperation. In the social-dilemma game, participants saw the faces of 40 interactants, all of which had the same sex as the participant. Twenty self-resembling faces (i.e., morphs of the participant) and 20 other-resembling faces (i.e., morphs of the same-sex yoked partner) were shown. Half of each type of interaction partner cheated, and the other half cooperated. The faces were randomly assigned to these conditions. Morphed photographs of yoked partners were used as control faces (instead of the original photographs) to avoid confounding self-resemblance with morphing. Otherwise, it would not have been possible to distinguish the effects of self-resemblance from effects of morphing. For instance, averaged faces are known to be perceived as more attractive [45]. Therefore, it is possible that morphed faces are associated with enhanced cooperation per se. Therefore, morphed pictures were used in both the experimental and the control condition to control for effects of morphing.

**Figure 3:**
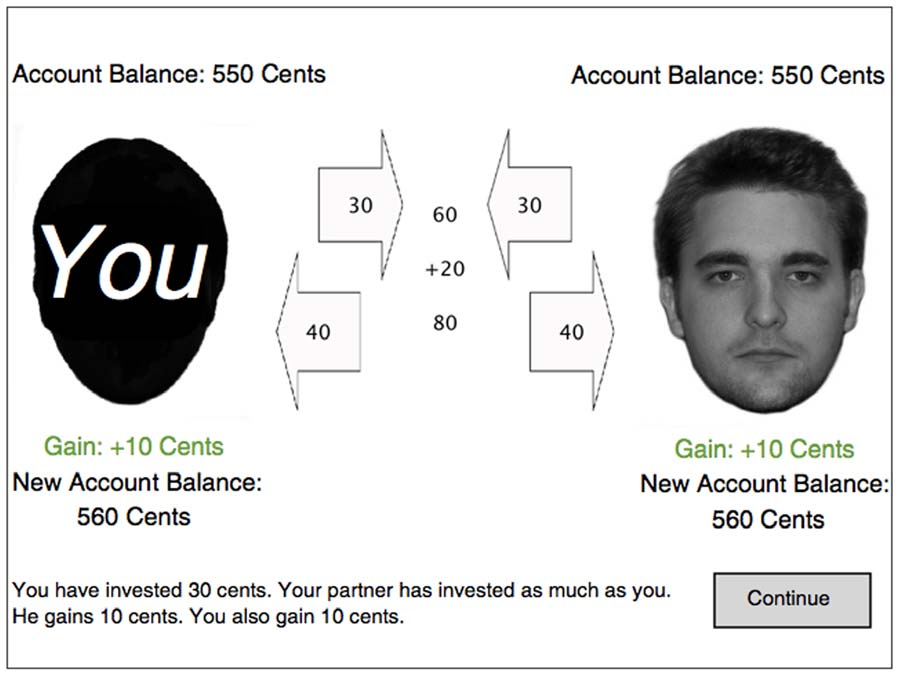
Screenshot of the social-cooperation game. On the left side, the participant is represented by a black silhouette. On the right side, a self-resembling or other-resembling face is shown representing the interactant. The number in the upper arrows refer to the investments. In this example, the interactant is cooperative and invests the same amount of money as the participant (30 cents). In the center of the screen, the sum of the investments and the bonus are shown. The numbers in the lower arrows refer to the participant's and the interactant's share of the total sum.

Each trial started with the presentation of a black silhouette on the left side of the screen representing the participant, and the face of the interactant on the right side of the screen (Figure 3). Above the participant's silhouette, his or her current account balance was shown. At the beginning of the game, the account balance was 550 cents. The participant was required to decide whether to invest 15 or 30 cents into a joint business venture with the interactant. Once the investment was selected and confirmed, the selected amount was shown in an arrow appearing on the upper right corner of the participant's silhouette. After 600 ms, the arrow moved towards the center of the screen (within 600 ms). Next, the interactant's investment was shown in an arrow appearing on the upper left corner of the interactant's face. Whether the participant made or lost money depended on whether the interactant cooperated or cheated. If the interactant was a cooperator, he or she reciprocated and invested as much money as the participant did (15 or 30 cents). If the interactant was a cheater, he or she refused to reciprocate and invested no money. The arrow with the interactant's investment moved to the center of the screen, where the sum of investments was shown. A bonus (⅓ of the sum of investments) was added, and the total sum was shown. In a last step, the total sum was divided between the interactants. Both received half of the total sum, regardless of what they had invested. The corresponding amounts were presented in two arrows at the center of the screen that moved towards the interaction partners at the left and right side of the screen, respectively. When the participant played with a cooperator, both interactants made money (5 or 10 cents, depending on the participant's investment). When the participant played with a cheater, the cheater benefitted at the expense of the participant. The participant had a loss that was as large as the gain in the cooperator condition (5 or 10 cents, depending on the participant's investment). On both sides, the amount of gain or loss (i.e., the difference between each interactant's initial investment and his or her share of the total sum) was shown. At the end of the experiment, the amount scored was paid out.

#### Source memory test

When the cooperation game was completed, participants received the instructions for the test phase. They saw a sequence of 80 faces, half of which had been encountered in the cooperation game, and half were new. Half of the new faces were self-resembling faces, and the other half resembled the yoked control partner. First, participants were asked to rate the likability of each face on a scale ranging from 1 (“not likable at all”) to 6 (“extremely likable”). Then, they were asked to indicate whether the face was old or new. When a face was classified as old, participants were asked to specify whether the face belonged to a cheater or to a cooperator. Then the next face was presented. It was randomly determined whether a face was used as the face of an interactant in the cooperation game or as a new face in the old-new face recognition test.

In a debriefing interview after the source monitoring test, participants were asked whether they noticed anything about the stimulus faces. Two of the participants reported that they had recognized their own face, as one would expect when manipulating facial self-resemblance near threshold. The two participants and their yoked partners were excluded from further analysis. Note that it is standard to manipulate facial resemblance near threshold [11], [25].

### Design

A 2×2 within-subjects-design was used with facial resemblance (self vs. other) and interactant behavior (cheating vs. cooperative) as within-subject factors. The dependent variables were the cooperation-game investments, the likability ratings, old-new face recognition performance, source memory performance, and the amount of benevolent guessing in the source memory test. Given a sample size of *N* = 58, 80 responses in the source memory test and *α* = .05, the power to detect a difference between the source guessing parameters for self-resembling and other-resembling interactants with an effect size of *w* = 0.06 (which is in the order of magnitude of the effects on guessing observed by Bell et al. [26]) was reasonably large (1–β = .98). The same applies to the general linear model within-subject comparisons. An effect of size *f* = .25 could be detected with a probability of 1–β = .95, assuming an average population correlation between the levels of the behavioral history repeated-measures variable of ρ = .6. The power calculation was conducted using G•Power [46].

## Results

**Figure 4:**
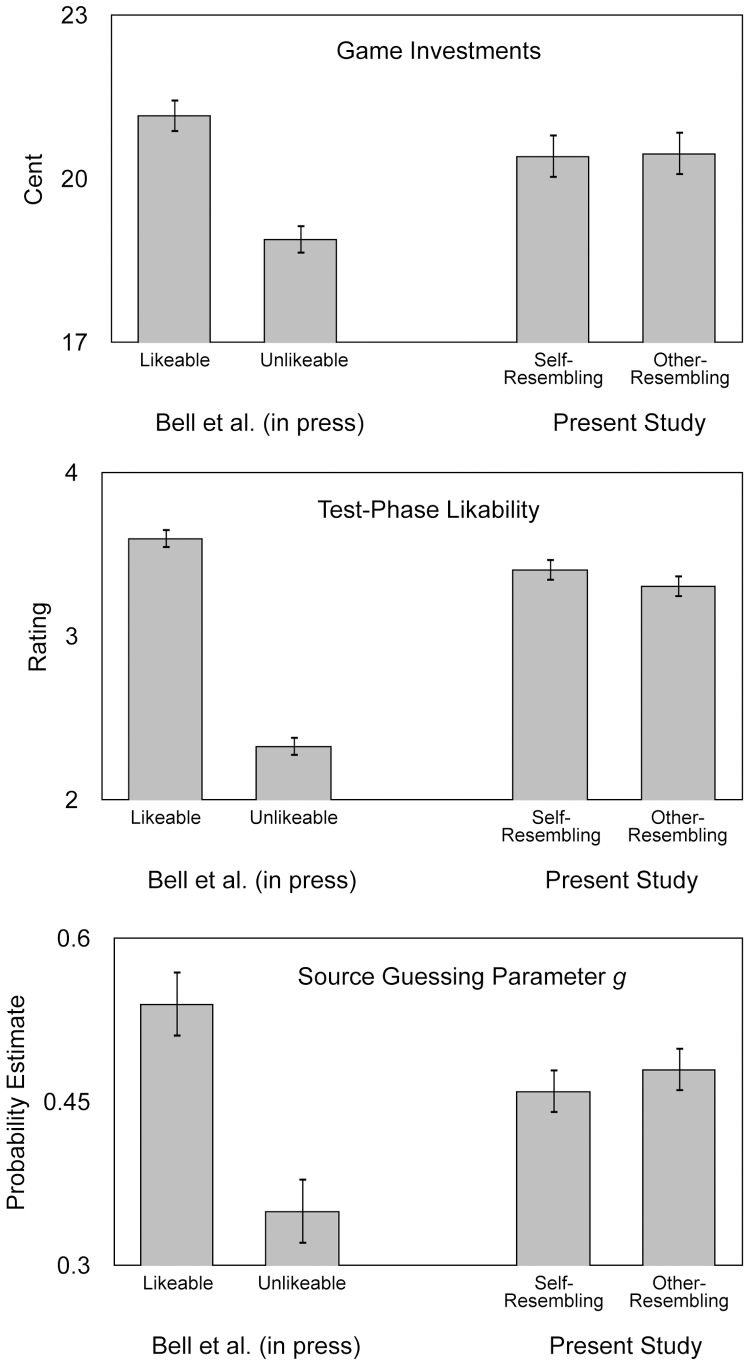
Cooperation-game investments, likability ratings, and the amount of benevolent guessing in the source memory test as a function of facial likability in Bell et al. 's (in press) Experiment 1 (for comparison only) and as a function of facial self-resemblance in the present experiment. Upper panel: Mean investments into the cooperation game. Participants could choose to invest 15 or 30 cents. Middle panel: Mean test phase likability ratings on a scale ranging from 1 (“not likable at all”) to 6 (“extremely likable”. Lower panel: Probability estimate of parameter *g*, which represents guessing that a face (of which the behavior was not known) belonged to a cooperative person. Thus, a high *g* probability estimate represents a tendency towards guessing that the face belonged to a cooperator, whereas a low *g* probability estimate represents a tendency towards guessing that a face belonged to a cheater. The error bars represent the standard errors of the means.

The results of the present experiment are displayed on the right side of Figure 4. For reasons of comparison, we display the results of Experiment 1 of Bell et al. [26] on the left side of Figure 4. In that experiment, we had manipulated facial likability by presenting faces of high or low facial likability that were selected based on consensus judgments. As can be clearly seen, all three dependent variables displayed in Figure 4 were significantly affected by facial likability in the previous study. These dependent variables were also used in the present study. We can thus be sure that the present paradigm is highly sensitive to manipulations of facial cues to trustworthiness.

### Game investments

**Figure 5:**
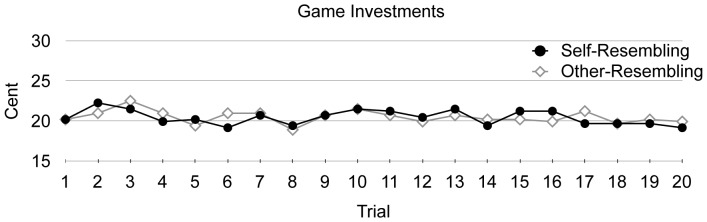
Mean cooperation-game investments for self- and other-resembling interactants in each of the 20 trials of the cooperation game.

The upper panel of Figure 4 shows the mean investments in the cooperation game. On the left side, the effects of facial likability (obtained in the previous study) are shown. As can be seen, facial likability had a pronounced effect on the participants' decision to invest into the cooperation game. Given that investing only makes sense when cooperation is anticipated, this finding is consistent with the idea that facial likability enhances trust. In contrast, as the right side of Figure 4 shows, there was no effect of self-resemblance on the investments, *F*(1,57) = 0.03, *p* = .87, *η* ^2^<.01. This finding is clearly inconsistent with the assumption that facial self-resemblance facilitates trust and cooperation. A trial-based analysis (Figure 5) showed that the game investments did not differ as a function of self-resemblance throughout the experiment. Thus, there was no difference between self- and other-resemblant faces even in the initial trials.

### Test-phase likability ratings

The center panel of Figure 4 shows facial likability ratings. Again, the left side shows the results of the previous study [26] in which faces with high a-priori likability received higher likability ratings than faces with low a-priori likability, consistent with the idea that there is a high level of agreement between individuals about the features that make a face likable or unlikable [28]. In contrast, in the present experiment, facial self-resemblance had no effect on the emotional evaluation of the faces as expressed in the likability ratings, *F*(1,57) = 1.51, *p* = .22, *η* ^2^ = .03. The likability ratings were descriptively lower for cheaters than for cooperators, *F*(1,57) = 3.28, *p* = .08, *η* ^2^ = .05. There was no interaction between self-resemblance and interactant behavior, *F*(1,57) = 0.01, *p* = .93, *η* ^2^<.01. Thus, the results indicate that there is not always a strong link between self-resemblance and the emotional evaluation of faces.

### Old-new-recognition

There was a main effect of self-resemblance on old-new-recognition in terms of the sensitivity measure of the two-high threshold (2-HT) model *P*_r_ [47], *F*(1,57) = 7.48, *p*<.01, *η* ^2^ = .12. Consistent with previous studies showing enhanced recognition of other types of self-resembling (own-race, own-age, and own-sex) faces [33], [34], [35], [36], participants recognized self-resembling faces (*M* = 0.44, *SE* = 0.03) better than other-resembling faces (*M* = 0.37, *SE* = 0.03). Consistent with previous studies showing no enhanced old-new recognition for faces of cheaters [48], [49], [50], [51], [52], old-new recognition was unaffected by interactant behavior, *F*(1,57) = 1.23, *p* = .27, *η* ^2^ = .02. There was no interaction between the two variables, *F*(1,57)<0.01, *p* = .96, *η* ^2^<.01.

### Measuring source memory

**Figure 6:**
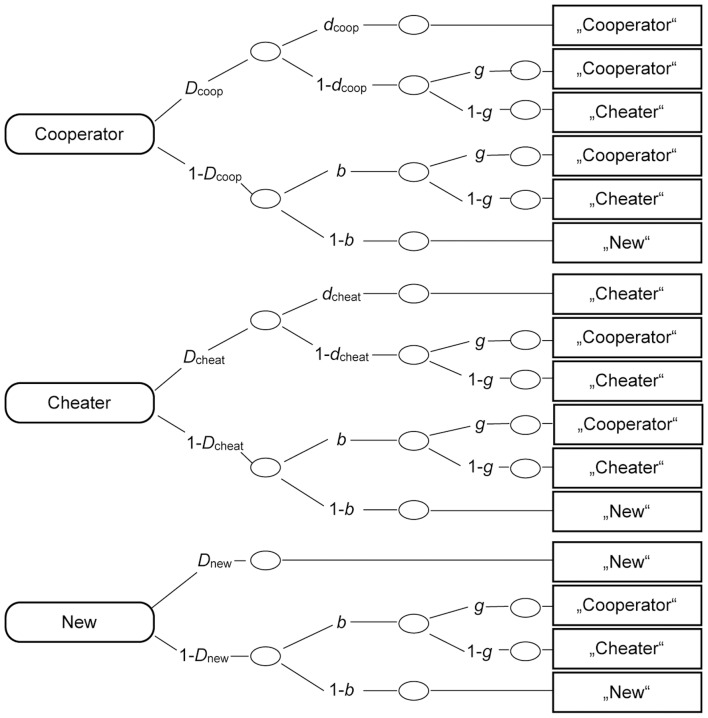
The source memory model adapted from Bayen et al. [53]. The rounded rectangles on the left side represent the stimulus category (cooperative, cheating, new). The rectangles on the right side represent the participants' responses to the faces in the source memory test. The letters along the links represent the probabilities with which certain cognitive states occur (*D*  =  probability of correctly recognizing a face as old or new; *d*  =  probability of correctly identifying the source of a face as cheating or cooperative; *g*  =  probability of guessing that a face belonged to a cooperative interactant; *b*  =  probability of guessing that a face was old).

To analyze performance in the source memory test, we used the multinomial 2-HT model of source monitoring proposed by Bayen, Murnane and Erdfelder [53] and displayed in Figure 6. This model is often used in source memory research [54] to separately assess the cognitive processes of old-new recognition, source monitoring and guessing which are assumed to underlie the observable classification performance in the source memory task. A huge advantage of this model is that validation studies have shown empirically that manipulations that are known to affect old-new recognition, source memory, and guessing, are accurately reflected, respectively, in the model parameters representing old-new recognition, source memory, and guessing [53], [55].

The first processing tree displayed in Figure 6 shows the processes that are assumed to occur when a cooperator face is presented at test. The probability *D*_coop_ represents the probability of recognizing the face as old. Parameter *d*_coop_ represents the conditional probability of remembering that the face is that of a cooperative interactant. With the complementary probability 1-*d*_coop_, it is not remembered that the face belonged to a cooperator. Then, the source has to be guessed. The source guessing parameter *g* represents the probability of guessing that the face belongs to a cooperative interactant. The complementary probability 1-*g* reflects the probability that a face is guessed to belong to a cheating interactant. When the face is not recognized as an old face with probability 1-*D*_coop_, it may still be guessed that the face is old with probability *b*. For these faces, it may be guessed that the face belonged to a cooperative interactant with probability *g*, or it may be guessed that the face belonged to a cheater with probability 1-*g*. With probability 1-*b*, the face is guessed to be new. The second tree represents the processing of the cheater faces in an analogous way. The last tree represents the processing of new (distractor) faces.

Please note that for reasons of simplification, only one set of trees is shown in Figure 6. To analyze the results of the source memory test adequately, we need two sets of these trees, one representing the self-resembling faces and one representing the other-resembling faces. Therefore, we also need two sets of parameters. For instance, there is one parameter representing the probability of guessing that a self-resembling face belongs to a cooperator, *g*_Self_, and one parameter representing the probability that an other-resembling face belongs to a cooperator, *g*_Other_.

### Source memory and guessing

The model shown in Figure 6 has more free parameters than can be identified based on the data, but it can be made identifiable by imposing appropriate restrictions on the parameters. Bayen et al. [53] provided a taxonomy of identifiable submodels of the source monitoring model. The most parsimonious identifiable model that still fits our data (Submodel 4 in the taxonomy of Bayen et al. [53]) includes the assumptions that old-new recognition and source memory do not differ between cooperators and cheaters (*D*_coop_  =  *D*_cheat_  =  *D*_new_; *d*_coop_  =  *d*_cheat_), which is consistent with the analysis of the old-new recognition data reported above and previous studies examining memory for cheaters and cooperators in social-dilemma games [27]. A discrepancy between these assumptions and our data would be reflected in the goodness-of-fit statistic *G* ^2^, which is asymptotically X^2^ distributed. However, the base model that included these assumptions fit the data well, *G* ^2^(4) = 3.12, *p* = .54, *w* = 0.03.

First, we tested whether the parameters representing item recognition could be set to be equal between self-resembling and other-resembling faces. As a restriction on the base model this assumption generates one additional degree of freedom. Self-resembling faces were better remembered than other-resembling faces, Δ*G* ^2^(1) = 6.95, *p*<.01, *w* = 0.04. This result confirms the analysis of the old-new recognition data reported above. Next, we tested whether the parameters representing source memory for self- and other-resembling faces could be set to be equal. Source memory did not differ between self- and other-resembling faces, Δ*G* ^2^(1) = 1.12, *p* = .29, *w* = 0.02.

We were most interested in testing whether guessing differed between self- and other-resembling faces. Specifically, previous studies have shown that facial likability, facial trustworthiness, and smiling were associated with an increased tendency towards guessing that a face was associated with cooperative behavior. It has been shown that these guessing biases are a good measure of trust and positive social expectations [26]. Based on the assumption that facial self-resemblance is associated with trust and positive expectations about the future social behavior of interactants, we expected that the guessing parameter *g* (reflecting the probability of guessing that a face belonged to a cooperative interactant if the true source of the face was not known) should be higher for self-resembling in comparison to other-resembling faces. Again, the left side of the lower panel of Figure 4 shows the effects of facial likability obtained in the previous study [26]. Clearly, the probability estimate of parameter *g* was higher for likable than for unlikable faces, which means that participants had a pronounced bias towards guessing that likable faces for which the true source was not known belonged to cooperators, and that unlikable faces belonged to cheaters. This finding is consistent with the idea that likable facial features are associated with positive expectations towards the cooperative behaviors of others [26]. In contrast, as the right side of the lower panel of Figure 4 shows, the guessing parameter *g* did not differ at all between self- and other-resembling faces, Δ*G* ^2^(1) = 0.67, *p* = .41, *w* = 0.01. Facial self-resemblance was clearly not associated with the expectation that the face belonged to a cooperator.

### Additional analyses

In two additional analyses, we evaluated whether the participants' sex, or their having siblings modulated the effects of resemblance on any of the dependent variables. A priori, it might have seemed possible that either female or male participants are more sensitive to the facial resemblance manipulation than male or female participants, respectively. It has been previously discussed that women may be more sensitive to facial resemblance in incest avoidance situations because they have to bear more costs when the offspring suffers from genetic defects than men [56]. Furthermore, it has been suggested that men are more sensitive to the self-resemblance of children's faces due to an adaptation that has evolved as a solution to the problem of paternal uncertainty [16], [17]. No gender effects are to be expected with respect to the effects of self-resemblance on cooperation, but a priori it seemed possible that sex-specific adaptations support the detection of facial self-resemblance and might therefore influence the results. Furthermore, it has been previously suggested that having siblings might enhance kin recognition in some situations (the effects have been demonstrated in a mating context, but not in an exchange-relevant context) [23]. However, the analyses revealed that the participants' sex and their having siblings had absolutely no influence on the self-resemblance effect.

The participants' sex had no main effects or interaction effects on any dependent variables, with one exception. There was a main effect of sex on the likability ratings, *F*(1,56) = 4.66, *p*<.01, *η* ^2^ = .21. Female faces received higher likability ratings by female participants than male faces by male participants. Although this effect should be interpreted with caution because of the comparably small sample of male participants, it might be due to the common finding that female faces are generally perceived as being more likable than male faces [57]. For the interpretation of the present findings, the effect can be considered irrelevant because there was no interaction between sex and facial resemblance.

Having siblings had no main effects or interaction effects on any of the dependent variables. However, there was a main effect of having siblings on the guessing parameter *g* in the source memory test, Δ*G* ^2^(2) = 9.37, *p*<.01, *w* = 0.04. Participants with siblings had a higher tendency of guessing that a face (of which the behavior was not known) belonged to a cooperative interactant than participants without siblings. This finding might suggest that people who grew up with siblings have more positive expectations towards the social environment. However, the effect should be interpreted with caution because of the small number of participants without siblings in our study. For the central research question of our study this main effect is irrelevant because there was no interaction with facial resemblance.

## Discussion

The data pattern found in the present study is clear and consistent. Consistent with previous studies showing enhanced recognition of self-resembling (i.e., own-race, own-sex, and own-age) faces [33], [34], [35], [36], facial self-resemblance had beneficial effects on old-new face recognition. This finding is most likely due to the fact that one's own face is a highly familiar stimulus with a particularly rich representation embedded in a large network [29], which may have beneficial effects on encoding and retrieval even when the participants are not aware of it. However, the hypotheses that were based on evolutionary theories of kin recognition were clearly not supported by the present study. Based on the assumption that facial self-resemblance is a kinship cue that stimulates kin support, we expected that facial self-resemblance should be associated with enhanced cooperation, likability, and trust. However, these predictions were clearly refuted by the results of the present study. (1) Facial self-resemblance did not affect the willingness to engage in cooperation. Participants did not invest more money into the cooperation game when the interactants' faces resembled their own face. (2) The emotional evaluation of the stimulus faces was not affected by facial self-resemblance in that self-resembling faces were rated as likable as other-resembling faces. (3) Facial resemblance did not affect trust in the sense of having confidence that the other person has cooperative intentions. The probability of guessing that the face belonged to a cooperator rather than to a cheater (when the true source of the face was not known) did not differ between self- and other-resembling faces. This guessing bias has previously been proven to be a good measure of positive emotional expectations in social contexts [26], [58]. In summary, our findings imply that self-resemblance does not lead to positive expectancies regarding the pro-social behavior of others, and does not trigger pro-social behavior towards others.

Before discussing the consequences of the present findings for theories about kin detection and facial resemblance, we discuss several methodological issues, and explain why we think that the present null results cannot be attributed to methodological problems. First, the null results cannot be attributed to low statistical power. Using 40 facial photographs of 58 participants should guarantee a sufficient reliability of the dependent measures and a sufficient statistical power to detect even small to medium effects [59]. Obviously, statistical reasons cannot explain the discrepancy to previous studies such as that of DeBruine [11] in which significant effects were obtained using eight facial photographs of 24 participants.

Second, when interpreting null effects, one faces the problem that this outcome could be either due to the absence of an effect of the independent variable or due to an insensitivity of the method used to measure the effect. To avoid this problem, we applied a paradigm that has been shown to be very sensitive to cues of trustworthiness in previous studies [26], [27]. For instance, high facial likability was associated with high investments in the social cooperation game, high likability ratings, and a tendency towards guessing that the person was associated with cooperative behavior (as shown in Figure 4). Likewise, the facial expression of the faces (smiling vs. anger), and facial trustworthiness had pronounced effects on these measures. Therefore, it seems noticeable that facial self-resemblance did not even lead to a consistent tendency towards cooperation, trust, and sympathy.

There is thus good evidence that the paradigm applied in the present study is sensitive to manipulations of facial trustworthiness. This rules out several alternative interpretations of the null effect. For instance, a potential concern is that our paradigm might have been insensitive to the effects of facial self-resemblance because participants did not believe to interact with real persons in the cooperation game. However, this hypothesis certainly does not explain the discrepancy to other studies in which effects of self-resemblance have been obtained in highly artificial laboratory situations [12], [13], [22], [23], and is inconsistent with previous evidence showing that the paradigm used here provides sensitive measures for detecting effects of facial trustworthiness [26].

Finally, one might be tempted to argue that the present null results could be explained by assuming that the facial phenotype matching mechanism is sensitive to other facial features than those manipulated in the present study. However, this interpretation is seriously challenged by the results of the norming study showing that the facial features manipulated in the present study clearly affected kinship judgments. The morphed stimulus faces and the faces of participants were rated as being even more closely related than actual first-degree relatives, which shows that self-resemblance was successfully manipulated. Given the results of the norming study, it seems quite plausible that a kin recognition mechanism should be activated by the manipulation used in the present study if it existed. Note that a popular hypothesis in Evolutionary Psychology is that people have evolved mechanisms that allow them to make accurate judgments about kinship based on facial features [15], consistent with the results of the present norming study. Furthermore, it has been speculated that the ability to recognize kin relationships between third parties might be a by-product of the capacity to detect one's own kin and relies on the same kin detection mechanism [15]. In contrast to these speculations, the present results show an interesting dissociation between the effects of facial resemblance on judgments of kin relationships in the norming study, and its lack of effect on trust and cooperation in the experiment. It is unclear why a kin recognition mechanism that is based on facial self-resemblance should be sensitive to other facial features than a kin recognition mechanism that is based on the resemblance between other people's faces.

Thus, the present study raises doubts about the robustness of the facial self-resemblance effect on trust and cooperation. It is noticeable that the paradigm used in the present study was shown to be highly sensitive to the effects of other cues of facial trustworthiness [26]. Furthermore, as our norming study shows, we used a strong manipulation of facial resemblance. Nevertheless, no effect on trust and cooperation was obtained. From this pattern of results, we conclude that the effects of facial resemblance on trust and cooperation are small and negligible in comparison to the large effects of other cues to facial likability and facial trustworthiness. Therefore, it seems questionable whether it is necessary to postulate a highly specific kin recognition module built into the face recognition system to explain effects of facial self-resemblance that seem to be only reliable in highly artificial laboratory tasks. In everyday life, there seem to be much more potent influences on cooperation that may mask an effect of facial resemblance. Therefore, we think that the fitness benefits associated with such a subtle and unreliable facial resemblance effect are too small to be selected for. Hence, it is questionable that the effects of facial resemblance on cooperation represents a highly specific adaptation built into the face processing system.

A possible interpretation of the overall pattern of results is that people do indeed show incest avoidance and enhanced cooperation towards close kin. This may have a subtle influence on the responses to kin-resembling faces that can be detected in strictly controlled tests. When seeing a face that resembles a close family member, people may show a transference effect, which is the well-known observation that we are influenced by the emotions towards our friends and relatives when we learn about new persons that resemble them or have something in common with them [14], [60]. This transference effect might be due to a general tendency to generalize our feelings and reactions. However, this effect may be so small and negligible in comparison to other influences on facial evaluation and cooperation that it can be only detected in highly artificial laboratory situations and is likely to be masked by much stronger influences on cooperative behavior in everyday life. If correct, then it is unlikely that the influence of self-resemblance on cooperation is strong enough to represent a highly specialized adaptation that has been selected for its beneficial effects on inclusive fitness.

Another possible interpretation of the present results would be to argue that the kinship recognition mechanism does not globally affect cooperation, but has rather restricted effects on cooperative behaviors. Our paradigm has been shown to accurately reflect the effects of variables (facial likability, smiling) that are commonly known to affect social perception and social behavior. However, it is possible that the beneficial effects of kinship on cooperation are not mediated by likability and trust. Given that cooperation with kin pays off in evolutionary terms even when the favors are not returned, one might cooperate with close relatives even when one does not expect that they will reciprocate. Therefore, one may expect to find more pronounced effects of kinship cues on measures of pure altruism (e.g., in a dictator game) than in the present paradigm that may be more closely linked to reciprocal altruism. Another interesting hypothesis is that people might only rely on phenotype matching in environments in which childhood coresidence and association to the mother are no reliable indicators of kinship (for example, when illegitimate children are frequent) [15]. In any case, the present results clearly show facial resemblance does not generally lead to detectable effects on all measures of trust and cooperation, as was claimed previously [11], [12]. The positive influence of facial self-resemblance on cooperation seems to be much more subtle and restricted in scope than previously thought.
